# Physicochemical Properties, Phenolic Acids Profile, and Vitamin Content of Quinoa‐Enriched Biscuits

**DOI:** 10.1002/fsn3.70368

**Published:** 2025-05-30

**Authors:** Ifra Batool, Tehseen Gull, Muhammad Abdul Rahim, Mohamed H. Mahmoud, Roberto Castro‐Muñoz, Eliasse Zongo

**Affiliations:** ^1^ Department of Chemistry Times Institute Multan Pakistan; ^2^ Department of Food Science & Nutrition Faculty of Medicine and Allied Health Sciences, Times Institute Multan Pakistan; ^3^ Department of Biochemistry College of Science, King Saud University Riyadh Saudi Arabia; ^4^ Department of Sanitary Engineering Faculty of Civil and Environmental Engineering, Gdansk University of Technology Gdansk Poland; ^5^ Laboratory of Research and Teaching in Animal Health and Biotechnology Bobo‐Dioulasso Burkina Faso

**Keywords:** antioxidants, *Chenopodium quinoa* Willd, nutritional quality

## Abstract

Quinoa ( *Chenopodium quinoa* Willd.) is a pseudocereal of great nutritional, pharmacological, and antioxidant value. It has the highest nutritional value of any food, including all fractions of amino acids, and is used as a complete protein food, especially for vegetarians and vegans. This work assessed the effectiveness of using quinoa‐flour fortified biscuits compared to wheat flour biscuits in terms of their physical appearance, chemical composition, antioxidants, phenolic acids, vitamins, microbial load, and sensory evaluation. Different types of treatment ratios of wheat and quinoa flour were considered: {0:100 (T_0_), 90:10 (T_1_), 80:20 (T_2_), 70:30 (T_3_), 60:40 (T_4_), and 50:50 (T_5_)}. The results indicated that the width and weight of the biscuits were significantly increased with increasing concentration of quinoa flour, while a significant decrease was observed in the biscuit diameter. Based on chemical composition values, quinoa fortified biscuits with the ratio (50:50) were acceptable as they contained higher protein content, fat content, fiber content, and ash content as compared to the control. Similarly, the content of minerals, total phenolics, total flavonoids, and vitamins increased as the concentration of quinoa flour increased in all treatments. Regarding the microbial count, quinoa fortified biscuits showed more resistance against bacterial and fungal colonization. Notably, Fourier transform infrared spectroscopy (FTIR) analysis revealed amines, esters, alcohols, and phenols functional groups responsible for the nutritional and sensory attributes of the biscuits. Therefore, sensory evaluation established high consumer acceptability, especially for biscuits with the ratio (50:50). This research work proves that fortification with quinoa adds nutritional value and may be stored for a long time without effect on sensory properties.

## Graphical abstract

Altogether, the result of this study supports the existing literature on the nutritional and functional properties of quinoa. The higher vitamin content, better nutritional quality, and higher antioxidant properties that were determined in biscuits fortified with quinoa are consistent with the positioning of quinoa as a nutrient‐dense and health‐benefiting ingredient. These findings provide the nutritional profile of quinoa, including being rich in high‐quality protein, vitamins and minerals, and bioactive compounds that have antioxidant and antimicrobial activities. The percentage of transmission and transmittance spectra from the FTIR analysis of the quinoa‐fortified biscuits shows the potential of quinoa in line with past studies on the functional and nutritive values of the seeds. Functional groups include amines, esters, alcohols, alkenes, phenols, and the like, which are found in quinoa and contribute to the health benefits and the texture of the biscuits. Quinoa fortification provides a viable strategy for improving both the nutrition and shelf life of the biscuits. This study supports the notion of quinoa as an ideal ingredient for food processing to improve nutritional qualities in foods. Although there are positive impacts related to diet, such as the nutritive values stemming from protein and minerals from quinoa, it is important to make the product worthwhile by retaining its favorable taste.

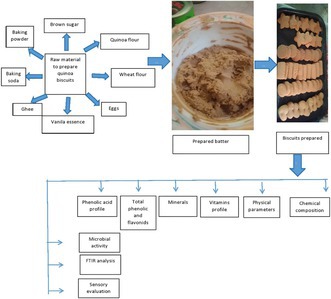

## 1 Introduction

*Chenopodium quinoa* Willd., an Andean pseudocereal of great nutritional, pharmacological, and antioxidant value, has the highest nutritional density of any food, which includes all nine fractions of amino acids and is used as a complete protein food, especially for vegetarians and vegans (Pathan and Siddiqui 2022). This protein complementation is between 14% and 18%, which is fairly close to the protein contained in animal‐based products (Camaggio and Amicarelli 2014). Besides this, quinoa contains dietary fats, which play an essential role in digestion and help regulate blood sugar levels. Being naturally gluten‐free, it is a good substitute for people suffering from celiac disease or gluten hypersensitivity (Melini and Melini 2021). In addition to protein, quinoa is also a rich source of minerals, including sodium, potassium, magnesium, zinc, iron, and phosphorus (Abd El‐Samad et al. 2018; Pathan et al. 2019; Adamczewska‐Sowińska et al. 2021). Nonetheless, quinoa contains many important nutrients, including vitamins B and E (Pirozi et al. 2017) and 89.4% unsaturated fatty acids especially omega‐3 fatty acids (Tang et al. 2015; Ng and Wang 2021; Pathan and Siddiqui 2022). Such nutritional qualities and vitamin and fatty acid profiles make quinoa a more nutritious food security choice that can serve different purposes across the different nutritional needs in society (Hernández‐Pinto et al. 2024; Castro‐Muñoz et al. 2024).

Phenolic acids are one of Quinoa's contributions to its antioxidant capacity yet to be fully understood. Phenolic acids belong to phytochemicals that have antioxidant activity and decrease the levels of oxidative stress, which participates in the development of chronic diseases (Tang et al. 2016). It was also observed from the study that quinoa is dominated by ferulic, vanillic, *p*‐coumaric, and gallic acids (Sidorova et al. 2022). These compounds contribute to the antioxidant capacity of quinoa due to their free radical scavenging activity and inflammation‐controlling activity in the body. Pharmacologically, quinoa contains bioactive compounds like saponins (Torres Vargas et al. 2021), flavonoids, phytosteroids, and phytosterols (Pathan and Siddiqui 2022), working as anti‐inflammatory, antihypertensive, and anti‐cancer agents (Agrawal et al. 2014; Simopoulos 2016). For example, there are so‐called saponins that are secondary metabolites accumulated in quinoa seeds and which have immunomodulatory and anti‐inflammatory effects (Vega‐Gálvez et al. 2010). It has also been proven that quinoa is able to control metabolic disorders such as diabetes because of its antihyperglycemic effect. Pasko et al. (2010) showed that extracts of quinoa have hypoglycemic effects in experimental animals. Consequently, its fiber content and low glycaemic index value qualify it as good food for diabetic patients or anyone with a risk of metabolic syndrome (Navruz‐Varli and Sanlier 2016).

Among these phenolic compounds, ferulic acid is the most significant nutrient present in quinoa seeds and has been surveyed for its antioxidant properties associated with the ability to inhibit lipid peroxidation and prevent cardiovascular diseases (Gil et al. 2021). Gallic acid and vanillic acid are also researched extensively for having antimicrobial and anti‐inflammatory effects. All these phenolic acids improve the nutritive value of quinoa as a functional food with potential health benefits (Tang and Tsao 2017). In sum, in benefit of its rich nutritional profile and its pharmacological and antioxidant characteristics, quinoa could be classified as a functional food. Moreover, the consumption of quinoa may provide the human body with proteins, fiber, and an appreciable amount of nutrients, vitamins, and minerals. It has also been reported that quinoa grains are good sources of bioactive compounds that can improve our immune system, safeguarding us from chronic diseases (Pathan and Siddiqui 2022). On these factual grounds, quinoa seeds flour can be supplemented in bakery products to prepare gluten‐free and highly nutritious edible products.

Cookies made with standard wheat flour are considered unsuitable for individuals who are suffering from celiac disease or gluten sensitivity (Biesiekierski et al. 2013). Whereas quinoa, being a gluten‐free grain with better nutritional quality than wheat, can be considered a good substitute (Elgeti et al. 2014). Additionally, quinoa‐fortified cookies may reduce the risks of gluten‐related issues, especially for gluten‐sensitive individuals. Moreover, the use of quinoa‐seed flour makes our food a rich source of amino acids, fiber, phenolic acids, flavonoids, and trace elements that make our diet healthy and nutritious (Pathan and Siddiqui 2022; Pirozi et al. 2017). Not only do these phenolic compounds offer antioxidant properties, but they also serve as quality preservers and as influential factors on the color, flavor, and texture of bakery products. Moreover, tocopherols (vitamin E derivatives) and flavonoids, which are present in quinoa, provide extra antioxidant activity to preserve the quality of fats in baked foods. This, in turn, favors the hypothesis that the fortification of quinoa improves the quality and shelf life of baked goods and has a positive impact on both the nutritional and functional characteristics of products (Hirose et al. 2010). If supplemented into bakery products, these bioactive compounds may assist in decreasing the rate of fat spoilage and thus increase shelf life (Tang and Tsao 2017).

There is no doubt that the researchers have worked on quinoa‐fortified bakery products, making pasta and bread (Hussein et al. 2023). Eliseeva et al. (2021) found that wheat bread enriched with quinoa flour showed an increase in protein, dietary fiber, thiamine, magnesium, iron, and phosphorus by 7%–11%, 5%, 16.2%, 17.8%, 24.5%, 16.7%, and phosphorus, respectively. Moreover, Hussein et al. (2023) found a remarkable increase in the nutritional quality of pan bread and pasta prepared from mixing quinoa flour with *Spirulina* algae powder. Besides these studies, very few research works are available that focused on preparing quinoa‐enriched biscuits, optimizing the proper ratio of quinoa‐seed flour and wheat flour. Secondly, a detailed profiling of quinoa‐enriched biscuits is also lacking in the literature. In the present investigation, quinoa‐enriched biscuits were prepared with the objective of optimize their ratio with wheat flour and study their physical and chemical attributes, sensory evaluation, nutritional quality, antioxidant potential, and profiling of phenolic acids and vitamins.

## 2 Materials and Methods

The ingredients used for the preparation of biscuits in the laboratory were quinoa seed flour, commercial wheat flour (protein 125 mg/g; an average particle size 10.15 μm) and all raw materials, including oil, brown sugar, eggs, baking powder, baking soda, and vanilla essence flavor, which were collected from local markets of Faisalabad and Multan, Punjab, Pakistan. All chemicals of analytical grade that were used in this study were purchased from Multan, Punjab, Pakistan.

### 2.1 Preparation of Quinoa Flour

After the collection of quinoa seeds, these were washed and rinsed with running water until the saponin layer was removed, as it gives a bitter taste to quinoa products. After drying the seeds, these were ground in an electric grinder to 70 mesh size, and this flour was used further to fortify biscuits.

### 2.2 Preparation of Quinoa‐Enriched Biscuits

Quinoa‐seed flour and wheat flour were mixed in 5 ratios. T_0_ was taken as control, containing 100 g of wheat flour with no quinoa (0:100). T_1_ contained 90 g wheat flour and 10 g quinoa flour (90:10) while T_2_, T_3_, T_4_, and T_5_ contained 80 g wheat flour and 20 g quinoa flour (80:20), 70 g wheat flour and 30 g quinoa flour (70:30), 60 g wheat flour and 40 g quinoa flour (60:40), and 50 g wheat flour and 50 g quinoa flour (50:50), respectively.

Before preparing quinoa‐enriched biscuits, the oven was heated to 180°C. Besides this, quinoa seed flour and wheat flour were added to a mixing bowl, and this mixture was set aside. The other ingredients, including oil, brown sugar, wheat flour, eggs, baking powder, baking soda, and vanilla essence flavor, were mixed in another bowl using a beater until a smooth and creamy consistency was achieved. After this process, this creamy mixture was slowly and gradually added to the flour mixture until a soft dough was prepared. After the dough was prepared, biscuits were made and baked in the pre‐heated oven for 5–7 min at 180°C until they turned golden brown (Davidson 2023). After completion of the baking process, these biscuits were stored at room temperature (25°C) and 50%–70% relative humidity for 4 weeks.

### 2.3 Physical Parameters of Quinoa‐Enriched Biscuits

The width of the biscuit samples was determined by using the Vernier Caliper (Victor 5150 digital Vernier Caliper). The diameter of biscuits was measured by using the scale (Libra scales). The spread ratio of quinoa‐enriched biscuits was determined by taking the diameter to width ratio using (Equation 1) below:

(1) $$\text{Spread Ratio}=\frac{\text{Diameter}\;}{\text{width}}$$

The weight of the samples was measured using a weighing balance (SF–400A, The Stationers, London, UK). Three values were taken to get an average. All these parameters were measured by using AACC (2000) method.

### 2.4 Chemical Composition of Quinoa‐Enriched Biscuits

The protein content of quinoa‐enriched biscuits was estimated using the Kjeldahl method 64–50 (AOAC 2006). Fiber content was determined using method no. 978.10 of AOAC (2006). Further, fat was estimated using method No. 30–10 of AACC (2000). The moisture content of quinoa‐enriched biscuits was measured using the official AOAC method 930.15 (AOAC 2006). On the other hand, samples of quinoa‐enriched biscuits were placed in an electric muffle furnace (FHX–12, Daihan Scientific, Largo, FL, USA) to measure the ash content at ~ 500°C–550°C for 6 h (Method 08–01) and the concentration of carbohydrate was calculated by subtracting the amount of moisture, ash, fiber, and fat content from hundred (AACC 1999).

### 2.5 Mineral Analysis of Quinoa‐Enriched Biscuits

The mineral content of quinoa was determined using the AACC (2000) method on an atomic absorption spectrophotometer (Scitek Global model SP‐AA4530). For mineral analysis, one hundred and ninety‐five grams of the quinoa‐enriched biscuits sample was put in a glass tube. A digestion solution composed of 65% nitric acid and 70% perchloric acid was digested further at 200°C to ensure that the epitome of the sample was perfectly homogeneous, since the clearing of the solution and its transparency signify perfect digestion of the sample. When the solution was digested, it was filtered to determine the presence of minerals, including potassium, calcium, magnesium, iron, and zinc content using an atomic absorption spectrophotometer.

### 2.6 High‐Performance Liquid Chromatography (HPLC) Analysis

Quantitative determination of vitamins and phenolic acids in quinoa‐enriched biscuits was done using HPLC (Agilent Technologies 1100 Series system) to separate the liquid mixture into its components by passing through a solid column. A C‐18 column = 250 × 4.6 mm, 5 μm, flow rate of 1.0 mL/minute, UV/Visible spectrometer detector was used to detect the vitamins and phenolic acids of quinoa‐enriched biscuits. For separation, a binary solvent gradient system was employed while the diode array detector was tuned at 254 nm. With this configuration, it was possible to accurately determine the vitamins and the phenolic acids, which are present in quinoa‐enriched biscuits to support nutritional data and the usefulness of these products (Robards 2003; Miranda et al. 2011).

### 2.7 Vitamin Analysis

Vitamins, including vitamin riboflavin (B_2_), pyridoxine (B_6_), folic acid, cobalamin, vitamin E, and β‐carotene, were extracted according to the standard method of AACC (1999), with some modifications. For estimation of vitamin B groups, 2 g of sample was poured into 0.1 N sulfuric acid solution (25 mL) and kept in a dark place at 121°C for half an hour. After heating, the mixture was cooled to room temperature. Then, 2.5 M CH_3_COONa and 50 mg Takadiastase enzyme were mixed well and stored at 35°C for 12 h. After incubation, the mixture was filtered through a Whatman No. 4 filter, and the solution was diluted with pure water (50 mL). For fat‐soluble vitamins, a 10 g sample was poured into the mixture of pyrogallic acid (1 g), ethanol (70 mL), and 50% KOH (30 mL). The mixture was stirred and refluxed for 40 min in a water bath at a temperature of 50°C. Then, the solution was neutralized using double‐distilled water, which was dehydrated using anhydrous sodium sulfate. The prepared solution was concentrated using a water bath at 50°C until 5 mL remained, and then the solution was diluted by the addition of methanol and stirred manually at room temperature. At the end, the solution was filtered using a 0.45 μm membrane to enhance clarity and avoid blocking up of the HPLC system (Miranda et al. 2011).

### 2.8 Phenolic Acid Analysis

For the quantification of phenolic acids, 0.5 g of quinoa‐enriched biscuit sample and 0.5 g of phenolic acids standard were taken in the flask. The extraction process was carried out in an ultrasonic bath, with 50% (v/v) methanol for 30 min. Subsequently, the mixture was centrifuged at 3000 rpm for 5 min after the extraction. The supernatant was filtered to remove any form of particulates in the sample before aspirating it into the HPLC system using a micro syringe (Robards 2003).

### 2.9 Quantification of Polyphenols and Flavonoids of Quinoa‐Enriched Biscuits

#### 2.9.1 Total Phenolic Content (TPC)

The total phenolic content of quinoa‐enriched biscuits was analyzed in absolute terms using a method developed by Singleton (1999) with Folin‐Ciocalteu reagent as the major analytical reagent (Ismail et al. 2017). First, 2.5 mL of 10% Folin‐Ciocalteu reagent was mixed with a solution of the quinoa‐enriched biscuits. Subsequently, 2 mL of 2% sodium carbonate solution was added to the mixture, and the reagents were well dispersed in the mixture by stirring. The solution was then allowed to stand for 30 min at room temperature for the differentiation of phenolic compounds that formed the color reaction. Finally, after incubation, the absorbance of the resultant solution was determined by using a UV/Visible spectrometer (Models: SP‐VG722, SP‐VGI721, SP‐VG7210, Scitek Global) at 765 nm. To determine the quantity of total phenolic content present, standard calibration curves were plotted using gallic acid at different concentrations, and water was used for preparing the standard solutions of the gallic acid.

#### 2.9.2 Total Flavonoid Content (TFC)

The total flavonoid content in the quinoa‐enriched biscuits was determined by the aluminium chloride colorimetric method (Žilić et al. 2011). A volumetric flask containing the biscuit sample was prepared as follows: 1 mL of biscuit sample was mixed with 4 mL of distilled water. To this mixture, 0.3 mL of 5% sodium nitrate was added and allowed to precipitate for 5 min. Subsequently, 10% aluminium chloride solution was added and after reacting for 6 min more, the mixture was filtered. Last, 2 mL of sodium hydroxide was put into the solution. Upon complete addition of all reagents into the solution, the total volume of the solution became 10 mL, and the solution was mixed for incorporation of reagents. The absorbance of the obtained solution was then read using a spectrophotometer at a 510 nm wavelength. For the estimation of the total flavonoids, calibration curves were made from different concentrations of quercetin, through which the sample absorbance values were compared for accurate quantification of flavonoid content.

### 2.10 Microbial Analysis of Quinoa‐Enriched Biscuits

#### 2.10.1 Total Bacteria and Fungi Count

AACC (2000) method was used to evaluate bacterial and fungal contamination levels within biscuit samples during the microbial load determination. 1 g of biscuit sample was aseptically blended with 9 mL of sterile 0.1% peptone water, thereby preparing a 10‐fold^−1^ dilution. Serial dilutions were then performed by transferring 1 mL from each dilution into 9 mL of sterile peptone water to generate the two dilutions of 10^−2^ and 10^−3^. Each dilution was thoroughly stirred to achieve equally dispersed microorganisms (AACC 2000). Samples from the prepared dilutions were placed on plate count agar before incubation at 37°C for 24–48 h as per International Organization for Standardization (ISO) 4833–1:2013 (ISO, E. 2013). Potato dextrose agar supplemented with chloramphenicol acted as the medium to enumerate yeasts and molds with plates incubated at 25°C for 3–5 days according to ISO 21527‐1:2008 (ISO 2008; Salfinger and Tortorello 2015). After incubation, plates with microbial counts from 30 to 300 colony‐forming units (CFU) were selected for proper counting. Microbial loads were recorded as CFU per gram of sample (CFU/g) following ISO 21527‐1:2008.

### 2.11 FTIR of Quinoa‐Enriched Biscuits

FTIR spectroscopy characterization was done using a Cary 630 FTIR spectrometer of Agilent Technologies over the range of 650–4000 cm^−1^. For the analysis, a small part of the sample was placed on a glass plate on which another glass plate was placed. When trying to form the best plate to make a good film for analysis, the top plate was rotated a quarter turn. To avoid the effect of water in the FTIR spectra, the time between background and sample measurements was minimized, and the light path was purged with dry air or high‐purity nitrogen. It was then positioned in the sample holder of the spectrometer for the glass plate assembly to be examined. The spectrum was recorded at 4 cm^−1^ to minimize errors in the identification of chemical functional groups in the biscuits prepared from quinoa flour. Analytical work based on this technique is quite useful to determine the molecular content and structural profile of food products, which may give some information on their chemical behavior or interaction (Abidi 2021).

### 2.12 Sensory Evaluation of Quinoa‐Enriched Biscuits

Sensory evaluation of quinoa‐enriched biscuits was conducted using the 9‐point Hedonic rating scale to assess overall acceptability. For this, twenty trained healthy individuals (45% male and 55% females), aged 22–28 years, with a good understanding of biscuit sensory attributes and the hedonic scale were selected as sensory panelists, based on their sensory perception, availability and motivation (Issanchou et al. 1995; Meilgaard et al. 2007). These selected participants were trained to familiarize them with tasting methods and quinoa‐enriched biscuits for effective evaluation (Issanchou et al. 1997). These panelists were instructed to focus on color, flavor, aroma, texture, appearance, and overall acceptability, rating from 1 “I extremely dislike” to 9 “I extremely like.” Before evaluation, a general focus group discussion was conducted to familiarize the panelists with the study's product and objectives. The three‐digit randomly coded quinoa‐enriched biscuits were presented on sample plates to the panelists to avoid biases. The biscuit samples, maintained at a consistent temperature (23°C ± 2°C), were presented to the panelists in a controlled environment (booth) where continuous lighting and temperature (25°C ± 2°C) were ensured (Jariyah et al. 2024). Additionally, prepared mineral water was used to neutralize the taste buds and remove the aftertaste.

### 2.13 Statistical Analysis

The statistical evaluations of the data were done using Statistix version 8.1 analytical software. The biscuits' samples were tested three different times to get the most credible results. The analytical data were expressed as mean ± standard deviation (SD); SD is a well‐known measure that reveals variability and consistency of the data. Reporting data in this way allows for a better understanding of the central tendency (mean) and the spread (variability) of the experimental results, which is essential for interpreting the impact of quinoa fortification on biscuit quality.

## 3 Results

### 3.1 Physical Properties

The physical properties of the quinoa‐enriched biscuits are presented in Table 1. The incorporation of quinoa flour resulted in an increase in width and a decrease in diameter, compared to samples fortified with the same levels of quinoa flour. Maximum biscuit width (1.67 ± 0.23 cm) was recorded when T_5_ biscuits were prepared with equal wheat flour and quinoa flour ratios (50:50). However, the lowest width (1.16 ± 0.04 cm) was found in T_0_ and T_1_ formulation (90:10). Results showed that the T_0_ treatment resulted in 6.33 ± 0.18 cm biscuit diameter while the T_5_ formulation had 4.76 ± 0.14 cm diameter. It was further noted that the spread ratio of biscuits also declined when biscuits contained higher amounts of quinoa flour. The spread ratio reached its maximum value of 5.40 ± 0.03 in T_0_, while the least spread ratio (3.33 ± 0.11) was found in T_5_ biscuits. Moreover, a significant increase in the weight of biscuits was observed with the addition of quinoa flour, as T_5_ achieved the maximum weight of 12.65 ± 0.53 g while T_0_ had the lowest weight of 8.99 ± 0.32 g.

**TABLE 1:** Physical parameters of quinoa‐enriched biscuits.

Treatments	T_0_	T_1_	T_2_	T_3_	T_4_	T_5_
Width (cm)	1.16 ± 0.04^c^	1.16 ± 0.04^c^	1.19 ± 0.04^c^	1.27 ± 0.04^bc^	1.40 ± 0.04^b^	1.67 ± 0.23^a^
Diameter (cm)	6.33 ± 0.18^a^	5.70 ± 0.17^b^	5.40 ± 0.03^bc^	5.30 ± 0.21^c^	5.27 ± 0.12^c^	4.76 ± 0.14^d^
Spread Ratio	5.40 ± 0.03^a^	5.03 ± 0.15^ab^	4.46 ± 0.16^b^	4.42 ± 0.15^b^	3.67 ± 0.12^c^	3.33 ± 0.11^c^
Weight (g)	9.09 ± 0.32^a^	9.1 ± 0.29^a^	9.11 ± 0.34^a^	9.29 ± 0.41^a^	9.33 ± 0.49^a^	9.41 ± 0.50^a^

*Note:* T_0_, Control; T_1_, 90 g wheat flour and 10 g quinoa flour; T_2_, 80 g wheat flour and 20 g quinoa flour; T_3_, 70 g wheat flour and 30 g quinoa flour; T_4_, 60 g wheat flour and 40 g quinoa flour; T_5_, 50 g wheat flour and 50 g quinoa flour; Different superscript letters in a row indicate statistical differences among treatments at *p* < 0.05.

### 3.2 Chemical Composition

The level of quinoa flour also affected the biscuits' chemical composition. A positive relationship was found between protein content and quinoa flour supplementation levels; T_5_ had the highest protein amount of 24.53% ± 0.04% and a control of 7.77% ± 0.12%. Based on this result, it can be stated that quinoa flour is a good source of protein, thus, it can act as a good biscuit fortificant. The fat content was also improved by increasing the concentration of quinoa flour in the biscuits. In T_5_, the fat was 8.75% ± 0.33% while in the control sample, the fat was 4.96% ± 0.11%, respectively. Quinoa‐enriched biscuits prepared from T_5_ formulation showed the highest total dietary fiber content (3.23% ± 0.12%), while the lowest (1.59% ± 0.20%) was identified in the control. On the other hand, moisture content variation was relatively small; T_5_ moisture content was 8.43% ± 0.28%, and T_0_ was slightly higher at 10.1% ± 0.47%. There was an increase in ash content, which is attributed to the mineral content and T_5_, with the highest ash content of 2.63% ± 0.10% from the control of 1.13% ± 0.03%. Moreover, T_0_ had 70.2% ± 2.4% carbohydrates while T_5_ had the least at 54.93% ± 1.83% (Table 2).

**TABLE 2:** Chemical composition of quinoa‐enriched biscuits.

Treatments (%)	T_0_	T_1_	T_2_	T_3_	T_4_	T_5_
Protein	7.77 ± 0.12^c^	21.46 ± 0.83^b^	23.64 ± 0.94^ab^	24.31 ± 0.72^a^	24.44 ± 0.92^a^	24.53 ± 0.04^a^
Fat	4.96 ± 0.11^d^	6.54 ± 0.19^c^	7.94 ± 0.31^b^	8.55 ± 0.31^ab^	8.74 ± 0.28^a^	8.75 ± 0.33^a^
Fiber	1.59 ± 0.20^c^	1.60 ± 0.05^c^	2.45 ± 0.08^b^	3.04 ± 0.11^ab^	3.20 ± 0.10^a^	3.23 ± 0.12^a^
Moisture	10.1 ± 0.45^a^	8.36 ± 0.28^ab^	8.43 ± 0.33^ab^	7.07 ± 0.21^b^	6.17 ± 0.19^c^	6.32 ± 0.18^c^
Ash	1.13 ± 0.03^c^	1.51 ± 0.04^c^	1.67 ± 0.04^c^	2.33 ± 0.09^b^	2.43 ± 0.06^ab^	2.63 ± 0.10^a^
Carbohydrates	70.2 ± 2.4^a^	60.04 ± 1.8^b^	59.57 ± 2.08^b^	59.03 ± 2.36^b^	58.57 ± 1.87^b^	54.93 ± 1.83^c^

*Note:* T_0_, Control; T_1_, 90 g wheat flour and 10 g quinoa flour; T_2_, 80 g wheat flour and 20 g quinoa flour; T_3_, 70 g wheat flour and 30 g quinoa flour; T_4_, 60 g wheat flour and 40 g quinoa flour; T_5_, 50 g wheat flour and 50 g quinoa flour; Different superscript letters in a row indicate statistical differences among treatments at *p* < 0.05.

### 3.3 Mineral Analysis

The results of mineral analysis confirmed the increase in nutritional value of biscuits, which resulted from quinoa‐seed flour addition in recipes. An increase in essential minerals was recorded when the proportion of quinoa flour increased in the formulation. For instance, maximum potassium content (2954.7 ± 88 mg/kg) was exhibited by quinoa‐enriched biscuits when the biscuit was prepared with a 50:50 ratio of wheat to quinoa‐seed flour, while the minimal potassium content (100.12 ± 10.1 mg/kg) was found in the control (T_0_) treatment (Table 3). Likewise, the magnesium concentration of T_5_ reached 1022.3 ± 30.6 mg/kg while the control group displayed only 35 ± 3.6 mg/kg of magnesium content (Table 3). Calcium content of biscuits also exhibited a substantial increase when the maximum value of this content was recorded in T_5_ biscuits (526.33 ± 15.7 mg/kg), followed by T_4_ and T_3_, while the lowest calcium content was found in T_0_ in comparison to all other treatments. Similar trends were recorded in quantifying iron and zinc content, with T_5_ showing the highest content and T_0_ with the lowest iron and zinc content (Table 3).

**TABLE 3:** Minerals profile of quinoa‐enriched biscuits.

Minerals (g)	T_0_	T_1_	T_2_	T_3_	T_4_	T_5_
Potassium	100.12 ± 10.1^d^	2451.7 ± 68.6^c^	2735.7 ± 93^b^	2751.0 ± 90.7^b^	2923.7 ± 78.9^a^	2954.7 ± 88^a^
Magnesium	35 ± 3.6^d^	614 ± 17.8^c^	652 ± 20.8^c^	840 ± 23.5^b^	988.7 ± 33.6^a^	1022.3 ± 30.6^a^
Calcium	25.21 ± 2.4^d^	442.00 ± 13.2^cd^	461.33 ± 12^c^	498.67 ± 13.9^b^	514.00 ± 16.9^ab^	526.33 ± 15.7^a^
Iron	10.07 ± 1.7^d^	26.66 ± 0.8^c^	30 ± 0.7^bc^	35.33 ± 1.2^b^	41 ± 1.1^a^	45 ± 1.3^a^
Zinc	3.51 ± 0.5^e^	18.33 ± 0.4^d^	20.33 ± 0.5^c^	31 ± 1.1^b^	34 ± 1.1^ab^	37 ± 1.1^a^

*Note:* T_0_, Control; T_1_, 90 g wheat flour and 10 g quinoa flour; T_2_, 80 g wheat flour and 20 g quinoa flour; T_3_, 70 g wheat flour and 30 g quinoa flour; T_4_, 60 g wheat flour and 40 g quinoa flour; T_5_, 50 g wheat flour and 50 g quinoa flour; Different superscript letters in a row indicate statistical differences among treatments at *p* < 0.05.

### 3.4 Total Phenolic and Flavonoids Content

Significantly high increases in both TPC and TFC were obtained in the fortified quinoa flour biscuits as compared to the control (T_0_) sample. In this study, T_5_ had the highest TPC at 270.57 ± 8.11 mg/100 g compared to the T_0_ (57.21 ± 7.87 mg/100 g). The TFC also generally followed the same trend as that of the T_5_, with the value of 132.80 ± 4.24 mg/100 g, while the control had 2.44 ± 0.43 mg/100 g (Table 4).

**TABLE 4:** Total phenolic and flavonoid content of quinoa‐enriched biscuits.

Treatments	TPC (mg/100 g)	TFC (mg/100 g)
T_0_	57.21 ± 7.87^d^	2.44 ± 0.43^d^
T_1_	211 ± 6.33^c^	88.70 ± 3.01^c^
T_2_	233.37 ± 8.16^b^	103.13 ± 2.78^bc^
T_3_	252.30 ± 6.81^ab^	109.23 ± 3.16^b^
T_4_	254.33 ± 7.62^ab^	123.03 ± 3.69^ab^
T_5_	270.57 ± 8.11^a^	132.80 ± 4.24^a^

*Note:* T_0_, Control; T_1_, 90 g wheat flour and 10 g quinoa flour; T_2_, 80 g wheat flour and 20 g quinoa flour; T_3_, 70 g wheat flour and 30 g quinoa flour; T_4_, 60 g wheat flour and 40 g quinoa flour; T_5_, 50 g wheat flour and 50 g quinoa flour; Different superscript letters in a column indicate statistical differences among treatments at *p* < 0.05.

### 3.5 Phenolic Acids

In the study, the biscuits were also characterized for phenolic acids and flavonoids by using the HPLC system. Total and individual phenolic compounds were significantly affected by quinoa flour addition: T_1_ had the highest amount of protocatechuic acid, 21.66 ± 0.58 μg/100 g, as compared to the control, 3.23 ± 0.13 μg/100 g (Table 5). Further, chrysin, another antioxidant, was significantly higher in T_5_ (13.30 ± 0.46 μg/100 g) as opposed to the control (2.13 ± 0.11 μg/100 g). T_5_ had 244.53 ± 07.33 μg/100 g, while the control had 108.23 ± 4.51 μg/100 g. T_5_ samples contained the highest levels of rutin and kaempferol: 54.66 ± 1.85 μg/100 g and 13.60 ± 0.140 μg/100 g, respectively, compared to the control group. Other known compounds, including apigenin, rosmarinic acid, cinnamic acid, and syringic acid, also rose with increased (Table 5).

**TABLE 5:** Phenolic acids of quinoa‐enriched biscuits.

Treatments (μg/100 g)	T_0_	T_1_	T_2_	T_3_	T_4_	T_5_
Protocatechuic	3.23 ± 0.13^e^	21.66 ± 0.58^a^	19.30 ± 0.55^b^	15.13 ± 0.51^c^	14.26 ± 0.42^c^	10.23 ± 0.33^d^
Chrysin	2.13 ± 0.11^d^	7.66 ± 0.22^c^	8.36 ± 0.25^bc^	9.26 ± 0.27^b^	9.73 ± 0.24^b^	13.30 ± 0.46^a^
Sinapic	108.23 ± 4.51^d^	185.47 ± 5.56^c^	203.43 ± 6.71^b^	211.03 ± 5.27^b^	232.60 ± 6.28^a^	244.53 ± 7.33^a^
Rutin	10.23 ± 0.33^c^	39.73 ± 1.07^bc^	41.50 ± 1.28^b^	47.70 ± 1.38^ab^	52.30 ± 1.56^a^	54.66 ± 1.85^a^
Kaempferol	4.65 ± 0.16^c^	7.33 ± 0.21^b^	9.30 ± 0.29^ab^	9.50 ± 0.31^ab^	12.76 ± 0.33^a^	13.60 ± 0.40^a^
Apigenin‐7‐ glucoside	1.23 ± 0.06^d^	7.13 ± 0.21^c^	7.40 ± 0.20^c^	10.23 ± 0.26^b^	13.76 ± 0.45^a^	14.20 ± 0.42^a^
Rosmarinic	176.23 ± 5.78^d^	275.03 ± 9.07^c^	322.33 ± 8.69^b^	342.60 ± 10.27^ab^	350.80 ± 11.57^a^	338.33 ± 10.14^ab^
Cinnamic	40.23 ± 1.33^e^	77.63 ± 1.94^d^	78.03 ± 2.02^d^	86.97 ± 2.34^c^	98.37 ± 3.24^b^	107.97 ± 3.23^a^
Apigenin	21.23 ± 1.73^c^	38.50 ± 4.15^bc^	42.60 ± 1.27^b^	43.56 ± 1.43^b^	47.53 ± 1.23^ab^	50.80 ± 1.72^a^
Caffic acid	10.33 ± 1.56^d^	29.20 ± 0.93^c^	35.36 ± 1.16^b^	43.36 ± 1.25^ab^	47.20 ± 1.32^a^	49.10 ± 1.42^a^
Syringic Acid	7.09 ± 0.71^d^	18.16 ± 0.49^c^	21.20 ± 0.61^b^	22.16 ± 0.66^ab^	26.33 ± 0.73^a^	27.80 ± 0.91^a^
Vanillic Acid	10.23 ± 0.73^d^	284.97 ± 8.5^ab^	262.40 ± 7.08^bc^	272.73 ± 9.27^b^	298.73 ± 9.26^a^	252.30 ± 6.30^c^
Ferulic Acid	—	3.20 ± 0.08^d^	3.33 ± 0.10^c^	3.53 ± 0.10^bc^	3.70 ± 0.09^b^	4.13 ± 0.13^a^

*Note:* T_0_, Control; T_1_, 90 g wheat flour and 10 g quinoa flour; T_2_, 80 g wheat flour and 20 g quinoa flour; T_3_, 70 g wheat flour and 30 g quinoa flour; T_4_, 60 g wheat flour and 40 g quinoa flour; T_5_, 50 g wheat flour and 50 g quinoa flour; Different superscript letters in a row indicate statistical differences among treatments at *p* < 0.05.

### 3.6 Vitamin Analysis

In the present investigation, significant variation was also observed in the vitamin profile of quinoa‐enriched biscuits. The findings indicated that higher amounts of quinoa‐seed flour in the biscuit composition increased vitamin levels. It was found that T_5_ showed the highest riboflavin (vitamin B_2_) content at 0.39 ± 0.01 μg/100 g, with the least value in the T_0_ (0.15 ± 0.02 μg/100 g). Furthermore, vitamin B_6_ also increased from 0.014 ± 0.005 μg/100 g in the control (T_0_) to 5.63 ± 0.16 mg in T_5_ (Table 6). The folic acid content showed a significant increase from 0.91 ± 0.07 μg/100 g in the control (T_0_) and reached 6.83 ± 0.20 μg/100 g in T_5_. It was a very interesting finding that cobalamin was not detected in T_0_ biscuits, while a substantial increase in its value was observed when the proportion of quinoa‐seed flour increased from T_2_ (0.16 ± 0.003 μg/100 g) to T_5_ (0.28 ± 0.008 μg/100 g), as shown in Table 6. The vitamin E content reached 2.50 ± 0.07 μg/100 g in T_5_ from its initial measurement of 0.07 ± 0.01 μg/100 g in the control group (T_0_). β‐carotene showed a varying trend as the maximum value was recorded in T_3_ biscuits (3.36 ± 0.09 μg/100 g) followed by T_4_ and T_5_, which were statistically at par with each other (Table 6).

**TABLE 6:** Vitamins profile of quinoa‐enriched biscuits.

Vitamins (μg/100 g)	T_0_	T_1_	T_2_	T_3_	T_4_	T_5_
Riboflavin (B_2_)	0.15 ± 0.02^e^	0.24 ± 0.06^d^	0.29 ± 0.08^c^	0.32 ± 0.01^bc^	0.34 ± 0.09^b^	0.39 ± 0.01^a^
Pyridoxine (B_6_)	0.014 ± 0.005^c^	4.66 ± 0.11^bc^	4.86 ± 0.12^b^	5.31 ± 0.13^ab^	5.31 ± 0.13^ab^	5.63 ± 0.16^a^
Folic Acid	0.91 ± 0.07^e^	4.26 ± 0.12^d^	5.20 ± 0.17^cd^	5.36 ± 0.15^c^	6.26 ± 0.21^b^	6.83 ± 0.20^a^
Cobalamine	—	0.16 ± 0.003^c^	0.19 ± 0.004^bc^	0.21 ± 0.05^b^	0.23 ± 0.07^b^	0.28 ± 0.008^a^
Vitamin E	0.07 ± 0.01^d^	1.49 ± 0.03^cd^	1.59 ± 0.04^c^	2.23 ± 0.06^bc^	2.30 ± 0.92^b^	2.50 ± 0.07^a^
B carotene	1.22 ± 0.03^d^	2.50 ± 0.04^cd^	2.64 ± 0.06^c^	3.36 ± 0.12^a^	3.20 ± 0.08^b^	3.23 ± 0.09^b^

*Note:* T_0_, Control; T_1_, 90 g wheat flour and 10 g quinoa flour; T_2_, 80 g wheat flour and 20 g quinoa flour; T_3_, 70 g wheat flour and 30 g quinoa flour; T_4_, 60 g wheat flour and 40 g quinoa flour; T_5_, 50 g wheat flour and 50 g quinoa flour; Different superscript letters in a row indicate statistical differences among treatments at *p* < 0.05.

### 3.7 FTIR Analysis

**FIGURE 1:**
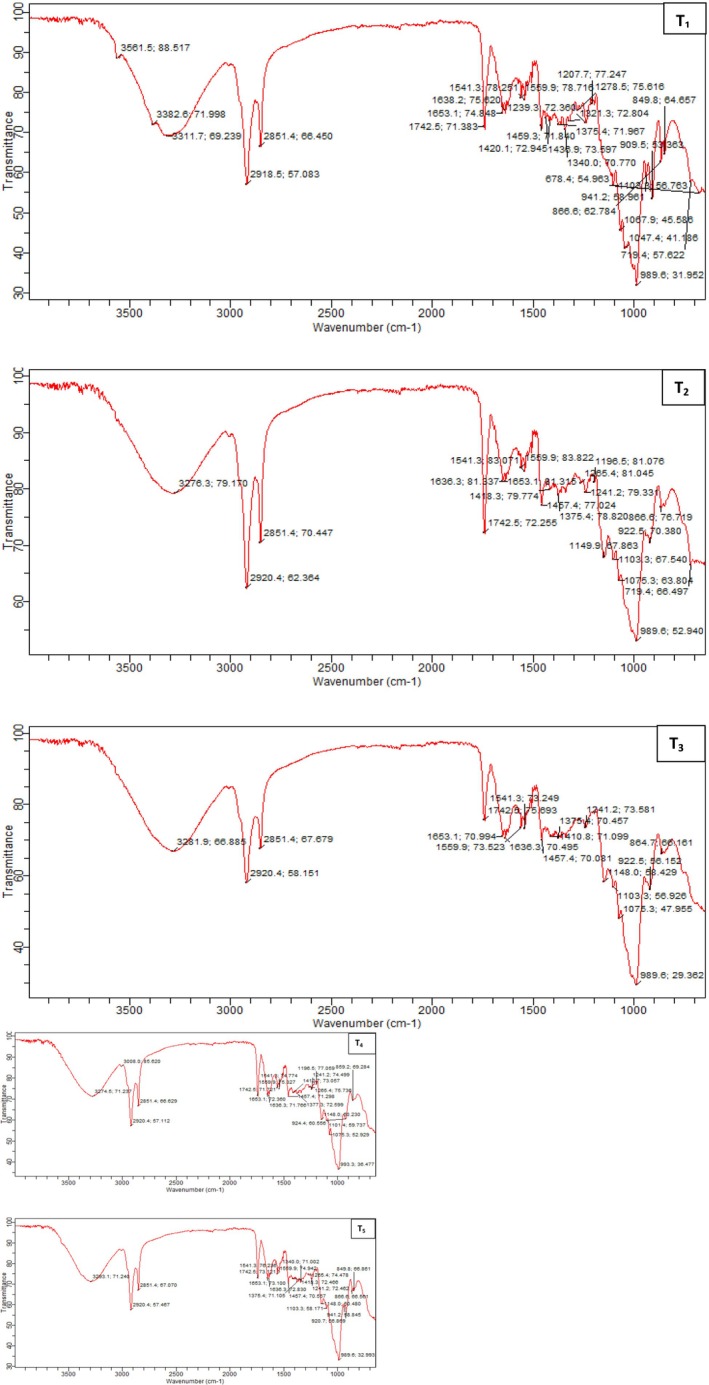
FTIR analysis of quinoa‐enriched biscuits.

The developing FTIR spectroscopy of quinoa‐enriched biscuits yielded molecular information on the structure of the biscuits and the functional groups presented in different biscuit samples, T_1_ to T_5_. As for the sample T_1_ of the FTIR spectrum, a broad peak was observed at 3382 cm^−1^ which points to the existence of an N‐H group and aliphatic primary amines. The last peak found at 1742 cm^−1^ was assigned to the carbonyl group of the carboxylic acid functional group. A second highest peak at 1239 cm^−1^ corresponds to the C‐O, which was an alkyl aryl ether group; another peak at 989 cm^−1^ corresponds to the alkene group (C=C) that shows unsaturated carbon‐hydrogen bonds. In the spectrum of sample T_2_, a broad peak was observed at 3278 cm^−1^ corresponding to the O‐H group. Alkanes and saturated hydrocarbons were identified at 2920 and 2851 cm^−1^ peaks. The peak at 1742 cm^−1^ indicates the existence of an ester moiety, while the peak at 1457 cm^−1^ (C‐H bond in alkane) and 1149 cm^−1^ is attributed to the C‐O molecule. In T_3_, a similar FTIR spectrum with a broad peak at 3278 cm^−1^ for the O‐H group (alcohol) and relatively sharp peaks at 2920 and 2851 cm^−1^ for C‐H bonds (alkane) was observed. The ester group (C=O) was present in the peaks recorded at 1742 cm^−1^ and the alkene (C=C) at 1653 cm^−1^. A symmetrical peak was observed at 1559 cm^−1^, which indicates the N‐H group. The characteristic of sample T_4_: there was a huge peak at 3278 cm^−1^, O‐H group (alcohol), C‐H bonds were present at 2920 and 2851 cm^−1^ of the alkanes bond. A peak at 1742 cm^−1^ shows an ester functional group, and the other peak at 1653 cm^−1^ reveals an alkene functional group. Thus, the peak at 1559 cm^−1^ attributed to an N‐H bond indicates the presence of an amine group. The band at 1457 cm^−1^ pointed toward C‐H bonds of alkanes, while the bands at 924 and 993 cm^−1^ pointed toward C=C bonds of alkenes. Finally, the characteristics of sample T_5_ included a broad peak at 3278 cm^−1^ related to the O‐H group (alcohol). The presence of C–H bonds consistent with alkanes was evidenced by the bands at 2920 and 2851 cm^−1^, and an ester bond (C=O) was shown at 1742 cm^−1^. Some of the characteristic peaks were observed at 1653 cm^−1^ for C=C, which indicates the presence of phenolic content. A peak at 1375 cm^−1^ supported the presence of phenol content and the O‐H group (Figure 1).

### 3.8 Microbiological Analysis

From the microbiological point of view, the biscuits containing quinoa showed better microbial loading than the control, thus compensating for this deleterious effect. There was no bacterial growth detected on fresh biscuits in all treatment groups. However, after 15 and 30 days of storage, superior to the control T_0_, which contained 17 × 10^5^ cfu/g at 30 days, T_2_, T_3_, T_4_, and T_5_ did not support bacterial growth. This indicates that quinoa flour may play a role in inhibiting bacterial growth, extending the shelf life of biscuits. Results obtained from fungi count revealed that T_2_, T_3_, T_4_, and T_5_ did not produce any fungi formation at all the storage days up to 30 days, whereas the control produced abundant fungi formation at 6 × 10^3^ CFU/g. (Table 7).

**TABLE 7:** Bacterial and fungi growth in fresh and stored quinoa‐enriched biscuits.

Treatments	Bacterial growth			Fungi growth		
	Fresh	15‐days storage	30‐days storage	Fresh	15‐days storage	30‐days storage
T_0_	0	3 × 10^5^	17 × 10^5^	0	2 × 10^3^	6 × 10^3^
T_1_	0	2 × 10^5^	21 × 10^5^	0	3 × 10^3^	4 × 10^3^
T_2_	0	2 × 10^5^	15 × 10^5^	0	0	0
T_3_	0	8 × 10^5^	20 × 10^5^	0	0	0
T_4_	0	5 × 10^5^	25 × 10^5^	0	0	0
T_5_	0	2 × 10^5^	19 × 10^5^	0	0	0

*Note:* T_0_, Control; T_1_, 90 g wheat flour and 10 g quinoa flour; T_2_, 80 g wheat flour and 20 g quinoa flour; T_3_, 70 g wheat flour and 30 g quinoa flour; T_4_, 60 g wheat flour and 40 g quinoa flour; T_5_, 50 g wheat flour and 50 g quinoa flour.

### 3.9 Sensory Evaluation

**FIGURE 2:**
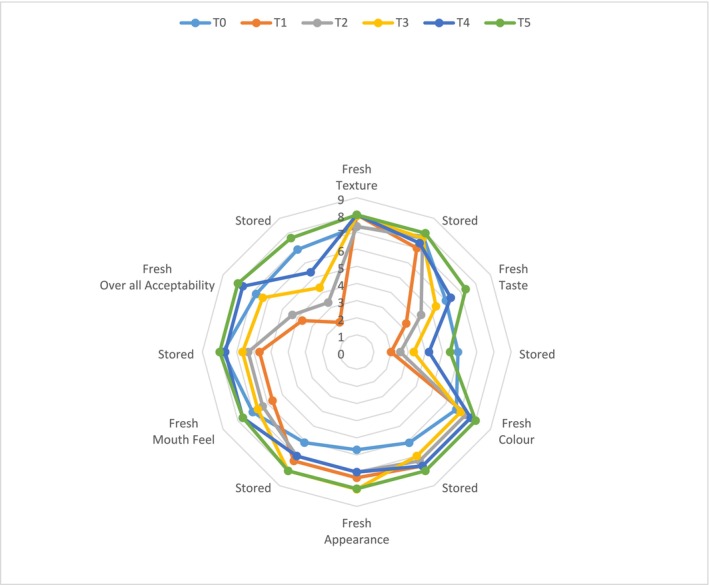
Sensory evaluation of quinoa‐enriched biscuits.

Concerning the acceptability of quinoa‐enriched biscuits, the findings of the present research displayed no significant difference in the overall acceptability of fresh and stored quinoa‐enriched biscuits. The acceptability scores of the biscuits prepared from quinoa and wheat flour blend lay between 2 and 7.67. Interestingly, the biscuits from the T_5_ biscuits were rated highest, while the T_1_ sample received the lowest rating (Figure 2).

To this extent, these findings indicate that biscuits with a relatively low level of quinoa incorporation are likely to be more acceptable than those with a high level of quinoa incorporation. Thus, from the present study, it can be observed that as the amount of quinoa used in the biscuit formulation is increased, consumers' preference for the biscuit is gradually reduced, though the improvement of biscuit quality.

## 4 Discussion

The findings on quinoa‐enriched biscuits in this investigation are therefore in conformity with earlier findings of the nutritional value of quinoa as a functional food ingredient. Many of the enhancements in vitamin content, physical alterations, and nutritional enhancement seen in this study stem from quinoa's natural inherent nutrient density and the confirmed role of quinoa in boosting the nutritional quality of a range of food items. The researchers have previously reported that diverse functional attributes of quinoa seeds make it a better supplement to fortify food products (Wang and Zhu 2016; Ng and Wang 2021). In the present investigation, quinoa‐enriched biscuits provided good results in terms of their abundance of phenolic acids, vitamin profile, and antimicrobial activities, which are in line with previous studies (Gil et al. 2021; Abdel‐Aal and Rabalski 2022).

To some extent, the outcome of this study on the issue of physical properties of the developed quinoa‐enriched biscuits follows the general trend of previous findings on the impact of quinoa flour on factors of spreadability, density, and structure of the products. When adding quinoa flour into biscuits, their width was enlarged while the diameter and spread ratio were reduced. This is in agreement with Schoenlechner et al. (2010) and Yeşil and Levent (2022) explanation that quinoa has comparatively higher proportions of protein and fiber, which influence dough resilience and moisture holding capacity. The lower spread ratio and diameter could be due to differences in gluten content; quinoa contained a lesser proportion of gluten than the wheat flour, which gave it the ability to expand as much as baked goodies (Daraz et al. 2020).

The effect of the increase in biscuit weight in relation to more quinoa flour agrees with the works of Vega‐Gálvez et al. (2010) who mentioned that the presence of quinoa in food items with high protein and dietary fiber consequently increases the density of the food product. This is seen in quinoa‐based bakery products and helps demonstrate the nutritional benefits of incorporating quinoa into this product despite reductions made to its texture characteristics.

The data obtained in this study demonstrated that the proposed method of fortifying biscuits with quinoa makes it possible to increase the contents of fiber and protein and also modifies other physical characteristics like spread ratio. The researchers have reported a direct correlation between protein and spread ratio, mentioning that the increase in protein might result in decreased spread ratio (Singh and Mohamed 2007; Yamsaengsung et al. 2012; Daraz et al. 2020). McWatters et al. (2003) reported that wheat flour‐based cookies possessed the highest spread ratio, but cowpea flour addition resulted in a decrease in this factor. It is reported that starch molecules in baking absorb water to achieve gel formation through gelatinization. This network structure displays its strength and rigidity based on the ratio between amylose and amylopectin components, together with the specific molecular characteristics of these polymers and their branched structures and lengths (Jackson 2003). During baking, the polymers, surrounding air and CO_2_ prevent them from escaping, which helps the expansion of dough while creating more crispness and puffiness in the baked product (Benjakul and Karnjanapratum 2018). Similarly, the spread ratio of quinoa‐enriched biscuits decreased with the increase in quinoa‐seed flour, which is also proportional to the protein content of the biscuits.

The findings of the present investigation manifested the increased protein, fiber, and fat content in the quinoa‐enriched biscuits, which proves quinoa as a good and nutritious substitute. Moreover, this study shows that protein has also risen exceedingly in batch T_5_ (24.53% ± 0.04%) compared to the control (4.96% ± 0.11%). The increase in protein content, along with the rise in fiber (3.23% ± 0.12% in T_5_), can be attributed to the higher content of protein, fiber, and carbohydrates in quinoa. Using data obtained from research conducted by Pritham et al. (2021), the protein content, fat, and carbohydrates found in quinoa seeds were 18.95, 5.44, and 57.88 g, respectively. Puri et al. (2020) also reported that quinoa‐fortified cookies prepared from a 60:40 ratio exhibited higher fiber content in comparison to wheat flour. In a previous study, Daraz et al. (2020) found a significant increase in protein and fat content with the increase of quinoa‐seed flour in supplemented cookies. They reported the highest protein content in cookies when these were supplemented with 50% quinoa‐seed flour, followed by 40% quinoa supplementation.

However, a rise in fat content from 10% in T_2_ to 8.75% ± 0.33% in T_5_ was also attributed to fats in quinoa being healthy fatty acids. The rise in quinoa‐enriched biscuits was also reported by Daraz et al. (2020). Moreover, Pathan and Siddiqui (2022) and Peiretti et al. (2013) have also reported quinoa seeds as a good source of crude fat and omega‐3 fatty acids. This fat profile improves the condition of the heart and arteries as it lowers low‐density lipoprotein cholesterol while raising high‐density lipoprotein cholesterol (Simopoulos 2016). Consuming quinoa as a dietary starch also provides fat calories and was found to have similar characteristics to the current study in that Pritham et al. (2021) have described quinoa as having high energy values (356.81 Kcal) owing to its macronutrient composition.

The assessment of the mineral contents of quinoa‐enriched biscuits demonstrates the extent of nutritional gains when quinoa flour is used to fortify biscuits. There was also a marked increase in potassium levels in T_5_ with 2954.7 ± 88 mg as against the control, which contains only 100.12 ± 10.1 mg of potassium, which adds to the testimony of quinoa being a great source of potassium that plays a great role in sustaining fluid balance, muscle build up, and transmission of nerve impulses (Fenn 1940). There was an increased and significant concentration of magnesium in T_5_ of 1022.3 ± 30.6 mg, while the control was 35 ± 3.6 mg, to show the importance of quinoa in muscle and nerve function, as well as an efficient component in biochemical romance in the body (Pohl et al. 2013). The fortification of wheat flour with chia and quinoa reportedly increased the calcium and iron content of fortified cookies (Goyat et al. 2018). Similar findings have also been found in the case of fenugreek‐fortified wheat biscuits (Hooda and Jood 2005).

Calcium also rose progressively with the addition of more quinoa flour; T_5_ was 526.33 ± 15.7 mg as compared to the control 25.21 ± 2.4 mg, establishing quinoa's place in bone health (Shin and Kim 2015). It also increased iron content; T_5_ contained 45 ± 1.3 mg, while the control contained 10.07 ± 1.7 mg, which is important for the synthesis of hemoglobin and oxygen transport in the body (Hirose et al. 2010). The levels of Zinc increased to 37 ± 1.1 mg in T_5_ from 3.51 ± 0.5 mg in the control; such results further support the proposition put forward in this research study that the process of fortification of quinoa can boost the immune system in humans since Zinc is well known to play a crucial role in immune response, wound healing, and protein synthesis (Ng and Wang 2021).

The present results support previous studies stating that quinoa is an adequate mineral source with high bioaccessibility. For example, Vega‐Gálvez et al. (2010); Yeşil and Levent (2022) discussed that quinoa contains a high concentration of requisite minerals like potassium, manganese, calcium, iron and zinc, which makes it a worthy ingredient for nutritional enhancement of food products.

Polyphenols are natural molecules acting as antioxidants that are commonly present in plants. They assist in preventing what is referred to as oxidative stress in the human body, a condition associated with many diseases, including heart diseases, cancers, and even diseases of the brain. Several epidemiological studies showed that a high intake of foods containing polyphenols may help lower the risk of these diseases (Agrawal et al. 2014). That said, adding quinoa to bread does more than improve nutritional value, but also ensures enhanced intake of polyphenols, which are usually lacking in the current diet.

Higher phenolic content and antioxidant capacity revealed in this research work add to earlier studies on the health benefits of quinoa bioactive compounds. (Gil et al. 2021; Sidorova et al. 2022). In this study, protocatechuic, sinapic, and cinnamic were some of the highest phenolic acids identified at much higher levels in the quinoa‐enriched biscuits than the control. These compounds are well noted for their antioxidant and anti‐inflammatory effects. For instance, in quinoa‐enriched biscuits identified to contain higher levels of protocatechuic acid, which, based on research by Tang et al. (2016), the compound offers strong antioxidant ability and alleviates inflammation. The strong antioxidant activity can also be attributed to the presence of vitamins (Liu et al. 2008).

The contents of two flavonoids—rutin and kaempferol, which demonstrated increased levels in the fortified biscuits and have been linked to protective effects against oxidative stress and anticancer activity. In line with previous studies, our recent study investigated the potential benefits of quinoa's flavonoids, foucusing more on chronic diseases. Consequently, the TPC and TFC increased from 57.21 ± 7.87 mg/100 g and 2.44 ± 0.43 mg/100 g in the control to 270.57 ± 8.11 mg/100 g and 132.80 ± 4.24 mg/100 g in T_5_, respectively, due to the antioxidant quinoa. Such an increase is supported by Gil et al. (2021) who stated that the phenolic compounds analyzed were considered to be hugely responsible for the antioxidant capacity of quinoa; thus, they can be highly recommended and used as an ingredient in functional foods to reduce oxidative stress and improve human health (Ghafar et al. 2017). The researchers reported that quinoa‐fortified bread exhibited improved polyphenolic content in comparison to wheat flour bread. The researchers further reported increased antioxidant activity in fortified bread, which is a sign of improved phenolic content (Gil et al. 2021). In that research, a 7.6‐fold, 13‐fold, 50‐fold, and 64‐fold increase was recorded in ferulic acid, sinapic acid, *p*‐hydroxybenzoic acid, and quercetin content of fortified bread, while rutin was not detected in wheat bread by Gil et al. (2021). In the present investigation, a five fold increase was observed in rutin content in comparison to wheat flour biscuits. The increase in rosmarinic acid and kaempferol, subject to the supplementation of wheat flour with quinoa flour, is also in line with a previous study. Gil et al. (2021) reported a 435% and 1304% increase in rosmarinic acid and kaempferol when wheat bread was fortified with quinoa flour, respectively.

Another interesting data point in the sample T_5_ spectrum reveals the presence of a phenol group characterized by a peak at 1375 cm^−1^. Flavonoids and other phenolic compounds are becoming widely preferred for their antioxidant effects, preventing oxidative stress, which is the main cause of various chronic diseases, including cancer and heart disease. The presence of phenols in the quinoa‐enriched biscuits confirms the previous investigation revealing the high phenolic content in quinoa. Several other researchers, such as Tang and Tsao (2017) and Pirozi et al. (2017), further categorized the phenolic compositions of quinoa to embrace ferulic acid and flavonoids, including quercetin and rutin, which determine the antioxidant content of the food.

The present study provided useful information concerning the bio‐accessibility and availability of quinoa‐enriched biscuits as a functional food ingredient for improving polyphenol consumption and antioxidant levels in daily food intake (Tang et al. 2016). Pseudo‐cereal, such as quinoa, has been reported to be packed with appreciable amounts of bioactive compounds like polyphenols, flavonoids, and other antioxidant compounds. It is worth approaching the development of biscuits with the addition of quinoa as an adjustment in the quality of the popular food items.

The test results obtained in this study reflect the results demonstrated from the antimicrobial efficiency tests done on the quinoa fortified biscuits in aspects relating to bacterial and/or fungal activity. Earlier literature has pointed out that quinoa possesses saponin and other bioactive compounds that may reduce the growth of microbes. Since no important bacterial and fungal development occurred in the quinoa‐fortified biscuits after 30 days of storage (treatments T_2_ to T_5_), including reduced spoilage counts and conformance to acidity levels, the replacement of part of the flour with quinoa may extend shelf life and enhance food safety of baked goods. This is consistent with the findings of Torres Vargas et al. (2021), which identified that quinoa has antibacterial saponins that may partly explain the biscuits' increased shelf life in this study. In previous literature, flavonoids have been attributed to strong antimicrobial and antifungal activities. Bloor (1995); Martini et al. (2004) have reported that kaempferol, which was found in appreciable quantities in quinoa‐enriched biscuits, has strong antibacterial activity against Gram‐positive and Gram‐negative bacteria, as well as against the fungus *Candida glabrata*. Based on these findings, it can be argued here that the microbial stability and shelf life of quinoa‐enriched biscuits can be attributed to the presence of flavonoids, as these compounds are responsible for antifungal and antimicrobial activities (Salas et al. 2011; Park et al. 2017).

Quinoa is well known for its vitamin content; B‐vitamins, vitamin E, and β‐carotene were also found to have gone up in the quinoa‐fortified biscuits (Alvarez‐Jubete et al. 2009; Tang et al. 2015). In this study, all the dependent variables of riboflavin (B_2_), pyridoxine (B_6_), folic acid, cobalamin (B_12_), vitamin E, and β‐carotene were enhanced as the proportion of quinoa flour added. The above observations are in parallel with the earlier findings that have pointed quinoa out as being a richer source of vitamins than cereals like wheat or corn, etc. (Navruz‐Varli and Sanlier 2016). These researchers emphasized that quinoa contains bioavailable B‐vitamins such as riboflavin and pyridoxine, involved in energetic metabolism and neuroprotection (Hernández‐Ledesma 2019). The increase of these vitamins, especially pyridoxine and folic acid, in the biscuits proves the hypothesis that adding quinoa to food products can solve deficiencies in B‐vitamins, which are important in the production of cellular energy and metabolism (Shulpekova et al. 2021). These findings clearly show that the fortification of quinoa increases not only the macronutrient content of baked products but also the micronutrient content of the baked products compared to those produced from wheat flour only.

The variable that stands out is the increase in folic acid, which has shown improvement from 0.91 ± 0.07 mg in the control to 6.83 ± 0.20 mg in T_5_. Researchers in earlier studies have pointed out the role that folic acid plays, in particular during pregnancy, to avoid the growth of neural tube defects, besides facilitating DNA synthesis and repair (Reynolds 2002). Schoenlechner et al. (2010) also conducted the study, which revealed enhanced folate content in quinoa‐based pasta; therefore, quinoa can act as a folate source in functional food.

Cobalamin (B_12_), which was not detected in the control, was present in gradually higher amounts in the biscuits containing quinoa, justifying the propitious effect of quinoa in increasing the micronutrient density of food products (Alamri et al. 2023). However, quinoa on its own is not rich in B_12_ (0.23 ± 0.07 mg/100 g), and the presence of cobalamin in the fortified biscuits may be attributed to microbial synthesis during fermentation or reactions with the other constituents during baking, according to Ansari and Nambiar (2023) who investigated the impact of quinoa fermentation on nutritional benefits.

Similarly, the increases in vitamin E from 0.07 ± 0.01 mg to 2.50 ± 0.04 mg and beta‐carotene from 1.22 ± 0.03 mg to 3.23 ± 0.09 mg may be explained by quinoa's purported antioxidant capacity. Miranda et al. (2011); Repo‐Carrasco et al. (2003) showed that quinoa contains a wealth of vitamin E and carotenoids that assist the body in combating oxidative damage and combating free radicals found in cells. These antioxidants play important roles in skin, immune, and cell health and the prevention of diseases, including cardiovascular and cancer diseases. This study evidences that including quinoa flour in biscuits has benefits such as increasing the antioxidant value, hence making biscuits more suitable for health‐oriented consumers.

The result of the FTIR of quinoa‐enriched biscuits agrees and complements existing studies on the chemical and nutritional characteristics of quinoa, as well as its incorporated food products. Functional groups recognized in the biscuits include alcohols, esters, amines, alkenes, and phenols; these groups are important in providing a molecular basis, structural characteristics, and health effects of quinoa‐based foods. These results provide a background to previous findings about the nutritional properties of quinoa and the use of this grain as a primary ingredient in fortifying agents for improving the texture and crust of baked products (Cao et al. 2021).

Depicted here also is the absorption due to N‐H bonds as a result of amine, particularly aliphatic primary amines, which are representative of proteins and amino acids. The existence of amines in the biscuits is as expected, given the high protein content of quinoa, and these samples include T_1_ and T_3_. Earlier literature reviews have noted that quinoa is one of the best plant‐source proteins (Schoenlechner et al. 2010). Interestingly, quinoa is rich in all needed amino acids and contains lysine to a definite extent, in contrast to wheat and corn. The amines closely relate to protein content, and other studies involving quinoa, such as those by James (2009), indicated that quinoa has significantly higher quality protein and higher digestibility than traditional grains.

In fact, studies revealed that protein content in quinoa is not only of nutritional value, but also has an impact on the rheology of food systems. Protein is on the other hand involved in the textural profile of biscuits and other bakery products, dough formation, elasticity, and textural attributes. The results obtained from the FTIR analysis that identified amines in quinoa‐enriched biscuits imply that quinoa proteins are enveloped with fats and carbohydrates to form a stable matrix. These molecular interactions may play a role in determining the biscuit's texture as well as its overall structure and integrity; specifically, Schoenlechner et al. (2010) attributed proteins in quinoa for enhancing elasticity and the bakery character of gluten‐free pasta.

The fact that ester groups (C=O) have been identified in all the biscuit samples, including, for instance, T_2_, T_3_, and T_5_, serves to highlight the role of fats in the biscuit formation and taste. Esters are present in fats and oils and are considered the bodying agents, and are responsible for the mouth feel of bakery products. Esters have been detected in biscuits, and hence, the presence of quinoa flour, butter, or oil in biscuits is responsible for the fat content. This is in concordance with the previous studies about the lipid profile of quinoa, which have revealed that quinoa is endowed with a large amount of unsaturated fatty acids such as oleic and linoleic acids. Fat content is a key nutrient which, according to Vega‐Gálvez et al. (2010), accounted for the energy density and health benefits of quinoa, including heart health due to healthy fat.

The presence of esters indicated in the FTIR spectra strengthens the notion that quinoa fortification enhances the quality attributes of biscuits in terms of texture and richness. This is further supported by Pathan and Siddiqui (2022), who pointed out that the fat content in quinoa improves the taste and storage of food items. Besides detecting the presence of esters, the reaction indicates the inclusion of healthy fats in the biscuits and the function of such compounds in preserving the structure and humidity of the final product.

The appearance of broad peaks represented by hydroxyl (OH) confirmed the presence of alcohols; this information is visible particularly in spectra corresponding to T_2_–T_5_. The hydroxyl groups are found to be bonded with sugars, polysaccharides, and phenolic substances, which are present in large amounts in quinoa. Literature reviews have noted that quinoa is rich in dietary fiber, soluble fibers like beta‐glucans, which are responsible for water holding capacity of, and moisture in food items. The observation of the O‐H groups in the FTIR correlates with a study done by Repo‐Carrasco et al. (2003), who proved that quinoa contains polysaccharides and soluble fiber, which contribute to the functional character of quinoa in the food matrix.

These hydroxyl groups also contribute to the antioxidant property of quinoa. Abdel‐Aal and Rabalski (2022) stated that quinoa has phenolic compounds with free hydroxyl radicals that make it have a high antioxidant value. The FTIR results suggest that fortification of quinoa increases the level of these constituents, which can benefit the quality of the biscuits largely in terms of nutritional contribution as well as preserve the fats from oxidative rancidity, thus increasing its shelf life. Researchers further added that the baking process results in noticeable increases in the abundance of unbound phenolic acids, which eventually could enhance their bioavailability and bioactivity.

The FTIR analysis of the treated samples also showed the existence of alkene groups (C=C), especially in T_1_, T_2_, and T_5_. There are, for example, the unsaturated hydrocarbons known as alkenes found in unsaturated fatty acids that are believed to be capable of improving the health of human beings in aspects such as their ability to deal with cardiovascular diseases. The detection of alkenes in the quinoa‐enriched biscuits is also in line with the lipid composition of quinoa through providing unsaturated fatty acids such as linoleic acid and alpha‐linolenic acids, as shown by Tang et al. (2015). The incorporation of unsaturated fats from quinoa also improves the textural parameters of the developed biscuits, including better moisture retention (Wang et al. 2015). The presence of positive FTIR bonding for alkene groups correlates with improving the lipid composition of the biscuits to support the nutritional and sensory benefits of quinoa.

Sensory evaluation is an important factor in new product development, especially when it comes to foods, since it offers a first‐hand indication from the consumers on the sensory characteristics of the food, including taste, texture, appearance, and acceptability. When applied to quinoa‐enriched biscuits, the study showed that the sensory evaluation got overall acceptability scores ranging from 2 to 7.67. Regarding the sensory properties of the samples, the T_5_ sample, which contained more quinoa, was rated higher than the other samples, whereas T_1_, with the lowest quinoa content, was rated the least. Surprisingly, and perhaps more importantly, there was no significant difference, *p* > 0.05, in overall acceptability between samples formulated with fresh quinoa and stored quinoa. This may mean that storage did not play any role in influencing the consumers' preference for the stored biscuits, which is encouraging as it shows that the biscuits are shelf‐stable.

Reduced consumption of quinoa might be associated with changes in texture, flavor, or even mouth feel due to increased quinoa content. Several aspects may be seen as vital in determining the acceptability of the product at high quinoa levels. The taste associated with quinoa and the mouth feel it gives to baked products are important. Schoenlechner et al. (2010) also found that quinoa has a potent flavor and increased fiber content, which makes it modify the sensory attributes of products such as bread and biscuits in a manner that may not be appealing to consumers when incorporated at high levels.

## 5 Conclusion

Altogether, the result of this study supports the existing literature on nutritional and functional properties of quinoa. The higher vitamin content, better nutritional quality, and the higher antioxidant properties that were determined in biscuits fortified with quinoa are consistent with the positioning of quinoa as a nutrient‐dense and health‐benefiting ingredient. These findings provide the nutritional profile of quinoa, including being rich in high‐quality protein, vitamins and minerals, and bioactive compounds that have antioxidant and antimicrobial activities. The incorporation of quinoa flour in biscuits improved protein content, vitamins and minerals, bioactive compounds, antioxidant, and antimicrobial activities. The percentage of transmission and transmittance spectra from the FTIR analysis of the quinoa‐enriched biscuits shows the potential of quinoa in line with past studies on the functional and nutritive values of the seeds. Functional groups include amines, esters, alcohols, alkenes, phenols, and the like, that are found in quinoa, bringing in health benefits and the texture of the biscuits. Quinoa fortification provides a viable strategy for improving both the nutrition and shelf life of the biscuits. This study supports the notion of quinoa as an ideal ingredient for food processing for improving nutritional qualities in foods. Although there are positive impacts related to diet, such as the nutritive values stemming from protein and minerals from quinoa, it is important to make the product worthwhile by retaining its favorable taste at a perfect ratio of quinoa‐seed flour fortification.

## Author Contributions

Conceptualization, T.G.; methodology, T.G.; supervision, T.G.; software, M.A.R. and T.G.; validation, T.G.; formal analysis, I.B.; investigation, T.G.; resources, M.A.R.; data curation, M.A.R.; writing – original draft preparation, I.B. and M.A.R.; writing – review and editing, M.H.M., M.A.R., R.C.M., and E.Z.; funding acquisition, M.H.M., R.C.M., and E.Z. All authors have read and agreed to the published version of the manuscript.

## Consent

Informed consent in writing from all participants ensured that participants were fully aware of the nature of the research and any potential risks or benefits.

## Conflicts of Interest

The authors declare no conflicts of interest.

## Data Availability

All data is presented in the article, and raw data is available on request.
